# Popular Fallacies

**Published:** 1893-06

**Authors:** James Hills


### HALL'S
### JOURNAL OF HEALTH

TRUTH DEMANDS NO SACRIFICE; ERROR CAN MAKE NONE.

VOL. XL. JUNE, 1893. No. 6.

### POPULAR FALLACIES.

It is a great mistake that a morning walk or other form of exercise before breakfast is healthful; the malaria which rests on the earth about sunrise in summer, when taken into the lungs and stomach, which are equally debilitated with other portions of the body from the long fast since supper, is very readily absorbed, and enters the circulation within an hour or two, poisoning the blood and laying the foundation for troublesome diseases; while in winter the same debilitated condi-dition of these vital organs readily allows the blood to be chilled, and thus renders the system susceptible of taking cold, with all its varied and too often disastrous results.

I do not wish to dismiss the statement which I made, with a simple assertion.

The denial of what is almost universally considered a truth so palpable, as scarcely to admit of proof, may well challenge investigation.

Besides, I do not want the regular readers of this journal to have their memories crowded with abstract precepts and pithy saws about health; I desire them, on the contrary, to become masters of general principles, to know and to understand the reason of things; then, these things can be remembered without an effort, while the principle being known, a very varied application is easily made and practically observed, a striking example of which is given in previous issues of the JOURNAL, in reference to the prompt cure of poisons and bites and stings of insects and reptiles, by the employment of familiar articles of kitchen use.

What I shall say on the subject of morning exercise is intended to apply mainly to all sedentary persons, those whose employment is chiefly indoors. And here I will simply appeal to the actual experience of any sedentary reader if he has not before now noticed when he has been induced from some extraordinary reason to take active exercise before breakfast on some bright summer morning that he felt rather a less relish for his food than usual ; in fact, had no appetite at all, there was a certain sickishness of feeling with a sensation of debility by no means agreeable.

It will be said here, this was because it was unusual, that if followed up, these feelings would gradually disappear. If that is so it is but a negative proof, for the system naturally has an inherent resisting power called into action by hurtful appliances. A teaspoon of brandy will produce slight symptoms of lightness of head in some persons if taken before breakfast, but if continued, the same amount will, after a while, produce no appreciable discomfort ; the cases are precisely parallel, that a man gets used to drinking brandy is no proof that it does not injure him.

Another person will remind me that the early air of a summer's morning seems so balmy and refreshing, so cool and delightful, that it cannot be otherwise than healthful.

That is begging the question; it is a statement known by scientific observers to be not simply untrue, but to be absolutely false. It is a common observation in New Orleans, where I lived a number of years, by those who remained in the city during the raging of the yellow fever, that when the air of mornings and evenings appears to be unusually delicious, so clear and cool and refreshing, it is a forerunner of an increase of the epidemic. Like the deceitful Syren it destroys while it lures.

*If you want to convince any body of anything argue alone.*

Having delivered ourselves of this great and useful apothegm we will resume the thread of the argument, taking it for granted that the reader has not forgotten the subject, *a summer's morning walk.*

It sounds charmingly, it brings with its mere mention recollections so mournfully pleasing or associations so delightful, that we long for the realization, at least until "sun up" to-morrow, then what a change ! we would not give one half-awake good stretch, one five minutes second nap, for all the summer morning walks of a whole year.

Who does not feel that the *vis inertia* of the first waking moments of a June morning, is worth more than a dozen rambles before breakfast. I am for the largest liberty of enjoyment; I am not among the multitude of the weak minded folk, the negative sort of minds, to discard what is good to eat or drink, or enjoy, for no other reason that I can perceive, than that it is good, and cross is meritorious. One man says that tea is injurious; another Solomon avers that that coffee makes people bilious; a third, and he an author, has written a book to prove that if we eat wheat bread it will make our bones brittle and that if we live to get old at all the first time we fall we'll break to pieces like a clay pipe stem. Verily this is a free country, for if everybody is to be believed, we are free to eat nothing at all.

So I do not advise a denial of that most deliciously enjoyable entity, a summer morning's nap, because it is for the reasons I have named, more healthful than the so lauded "*exercise before breakfast.*" If you must remain in bed until breakfast or be out in the open air an hour or two before breakfast, on an empty stomach, then I say, as far as health is concerned, the nap is better than the exercise, for the incontrovertible reasons I have already given. It requires no argument to prove the impurity of a city atmosphere about sunrise and sunset, reeking as it must, with the odors of thousands of kitchens and cesspools, to say nothing of the innumerable piles of garbage which the improvident poor allow to accumulate in front of their dwellings, in their back yards and their cellars; any citizen may satisfy himself as to the existence of noisome fumes by a summer evening's walk along any of our by-streets; and although the air is cooler in the mornings, yet the more hurtful of these malaria saturate it. But of such a subtle nature are they, that the microscopic observation,no chemical analysis has as yet been able to detect, in an atmosphere thus impregnated, any substance or subsistence to which these deadly influences might be traced, so subtle is the poison, so impalpable its nature. But invisible, untraceable as it may be, its influence is certain and immediate, its effects deadly.

Some will say, look how healthy the farmer's boy is and the daily laborers, who go to their work from one year's end to the other by "crack of dawn!" My reply is, if they are healthy they are so in spite of these simple exposures; their simple fare, their regular lives, and their out door industry, give their bodies a tone, a vigor, a capability of resisting disease, which nullifies the action of malaria to a very considerable extent.

Besides, women live as long as men, and it cannot be said that they generally exercise out of doors before breakfast.

Our Knickerbocker ancestry! the very mention of them suggests fat! A double fatness in fact, fat as to body, and fat as to purse; if you catch hold of one of them, instead of getting a little pinch of thin skin as you would from a lean Yankee, you clutch whole rolls of fat, solid fat. What substantial people the real, identical, original old Knicks are! how long they live, too! expectant sons-in-law echo, sighingly, "*how long!*" In fact I do not recollect of their dying at all, at least as we do; they simply ooze out or sleep away. May we not inquire if there is not at least some connection between their health as a class, and the very general habit of the sons here, derived from their sires in fatherland, of eating breakfast by candle light? Another very significant fact in point is, that the French in the south are longer lived and suffer far less from the fevers of the country than their American neighbors! in truth, their exemption is proverbial, and as a class they have their coffee and boiled milk, half and half, with sugar, brought to their bedsides every morning, or take it before they leave the house.

It is not an uncommon thing for persons to go west to select a new home for their rising families never to return; "*took sick and died!*" This is the sad and comprehensive statement of the widowed and the fatherless, owing doubtless, in many instances to their traveling on horseback early in the morning, and late in the evening, in order to avoid the heat of the day.

*Many a traveler will save his life by taking a warm and hearty breakfast before starting in the morning, and by putting up for the night not later than sundown.*

It is of considerable practical inportance to answer the question, why more persons have died in "the States" from Isthmus fever than in California! Simply, because on their way out, their bodies are comparatively vigorous, and there is in addition a degree of mental and moral excitement which repels disease; but on the return, it is strikingly different; the body is wasted by hardship and privation, while the spirit is broken by disappointment, or the mind falls into a species of exhaustion when successful, from the long and anxious strife for gold; both causes operating, one to weaken the body, the other to take away all mental elasticity; it is no wonder that the whole man becomes an easy prey to disease. JAMES HILLS, M. D.

